# Point-of-interest recommender model using geo-tagged photos in accordance with imperialist Fuzzy C-means clustering

**DOI:** 10.1371/journal.pone.0317131

**Published:** 2025-03-07

**Authors:** Ali Asghar Salehi Solaiman Abadi, Keyhan Khamforoosh, Vafa Maihami

**Affiliations:** Department of Computer Engineering, Sanandaj Branch, Islamic Azad University, Sanandaj, Iran; Ningbo University, CHINA

## Abstract

Although recommender systems (RSs) strive to provide recommendations based on individuals’ histories and preferences, most recommendations made by these systems do not utilize location and time-based information. This paper presents a travel recommender system by integrating the Imperialist Competitive Algorithm (ICA) and Fuzzy C-Means (FCM) Clustering algorithm. Compared to similar studies, this recommender system takes into account more POIs, including location, number of visits, weather conditions, time of day, user mood, traffic volume, season, and temperature. The effectiveness and accuracy of the proposed method are assessed using the Flickr dataset, indicating that it is able to provide effective and accurate recommendations that are compatible with the user’s interests and the current status of his/her visit. Results showed that, precision and Mean Absolute Precision (MAP) in the proposed method have been grown 23.6% and 23.72% in comparison to Popularity Rank, 28.98% and 19.67% in comparison to Classic Rank and 18.66% and 19.67% in comparison to Frequent Rank methods. Also, Mean Absolute Error (MAE) index in proposed method has been improved 60.71%, 64.51% and 56% in comparisons to the Popularity Rank, Classic Rank and Frequent Rank methods respectively.

## 1. Introduction

The ever-increasing growth of information in today’s world, the increased number of online users, and the need to quickly provide users with correct information have made recommender systems (RSs) vital tools in electronic businesses, with the aim of removing information overload [1]. These systems are designed to attract customers and gain their trust by providing the best and most appropriate product recommendations that are compatible with their interests and tastes [2]. More and more studies are conducted on recommender algorithms. By developing intelligent systems that evaluate the user’s past behavior as well as similar users’ behaviors, companies provide the user with suitable suggestions and recommendations [1], including suitable job opportunities, favorite movies, videos, and Facebook friends. This study also deals with the application of RSs in the tourism industry, where recommendations should be made based on the time and location of the tourist’s presence. Considering the fact that the income obtained from the tourism industry and the use of ever-developing new technologies is on the same level as oil income, it becomes apparent that they should be given more attention [3].

Existing recommender systems often underutilize point of interest (POI) information, leading to suboptimal recommendations. This paper aims to develop an improved travel recommender system that delivers more accurate recommendations by considering various factors such as location, time, weather conditions, and user preferences. Given the burgeoning growth of the tourism industry and the importance of providing high-quality services to tourists, developing precise recommender systems in this domain is essential. Enhanced recommender systems can assist tourists in planning their trips and improve their overall travel experience. By leveraging recommendations tailored to their location, time, and personal preferences, tourists can receive the best visit suggestions. Researchers in this study have employed more sophisticated algorithms like ICA and FCM, enabling them to analyze complex data more effectively and produce more accurate results. Furthermore, instead of solely focusing on location data, this research considers additional factors such as weather conditions, user interests, traffic volume, and other parameters, thereby improving the accuracy of recommendations. Overall, this research seeks to enhance the quality of life and travel experiences by developing smarter and more precise recommender systems. Tourists used to waste a lot of time due to unfamiliarity with the destinations. These recommendations solve that problem[4,5].

RSs should have a high precision when providing recommendations to tourists, as using fuzzy logic with uncertainty and providing effective recommendations can be useful [6]. In this study, the combined use of the two algorithms, i.e., ICA and FCM, with the aim of providing a stronger processing, increases the precision of the recommendations provided by the system, compared to the use of classical clustering.

What differentiates the proposed approach of this study from those of similar studies that have only used temporal and spatial POIs [51,52] is the addition of the number of POIs (weather condition, user mood, traffic volume, etc.), which has brought the users closer in a cluster, thereby increasing the precision. In the proposed method, the service quality parameters, including precision, Mean Absolute Precision (MAP), and Mean Absolute Error (MAE) are measured and evaluated. The data from Flickr, one of the largest online image and video sharing sites, was used to evaluate the presented method.

Flickr is one of the largest online platforms for sharing public images and videos. The impact of geographic information on increasing the efficiency of tourism recommendation systems (RSs) is undeniable. This is because most tourist attractions are located in close proximity to each other. To provide more accurate recommendations, in addition to geographic tags, other features should also be considered as supplementary information. Features such as the number of visits to the place of interest, weather conditions, traffic volume, and user mood are used to increase the accuracy of the proposed method and provide more precise recommendations.

Based on these parameters, first, the cluster centers and the number of clusters are determined using the ICA algorithm, and then the membership degree in the clusters is determined for each location using FCM, which is a fuzzy algorithm. Therefore, for a tourist at a specific location, all places in the same cluster can be a suitable recommendation. However, places with a higher membership degree are better candidates.

In short, the research innovations include:

- In the proposed method, the ICA has been used to ascertain the quantity of clusters, while the random method has been used in similar studies.
- The FCM algorithm has been used for accurate clustering and POI modeling of the data.
- The location and time POIs have been used in similar studies. In the proposed method, in addition to the location and time POIs, the number of visits (popularity level), weather condition, user mood, traffic volume, season, and temperature have been added.
- The remaining sections of the study are structured as follows: Section 2 covers the basics of the research and introduces RSs, discusses POI categories and types, and presents open issues in this field. Section 3 reviews relevant studies in the field of POI RSs. Section 4 presents the proposed method. Section 5 elaborately presents the evaluation results of the proposed method. Finally in Section 6, the conclusion remarks and suggestions for future studies are provided.

## 2. Research basics

The POI travel RS introduced in this study is based on fuzzy clustering and imperialist competitive optimization algorithms. In this section, we investigate the concepts of RSs and POI data, followed by the FCC and the imperialist competitive optimization algorithms as the foundations and building blocks of the proposed method.

### 2.1. Recommender systems

RSs are systems designed to assist users in finding and selecting items they desire. However, it is a common problem that these systems cannot provide appropriate recommendations without access to sufficient and accurate information about the users and their desired items, such as movies, music, and books. Therefore, one of the fundamental objectives of RSs is to gather diverse information about users’ preferences and the available items in the system [1]. There are various sources and approaches to collect this information. One approach is explicit data collection, where the user explicitly indicates their interests, for example, by rating a piece of music. The other approach is implicit, which is more challenging to implement. In this approach, the system must identify the user’s preferences by monitoring and analyzing their behaviors and activities, such as their most frequently listened to music, visited pages, and contacts[7].

Apart from implicit and explicit data, certain systems utilize users’ personal information, such as age, gender, and nationality, to identify and offer recommendations to them. This category of information is known as statistical information, which aids in creating a set of recommendation systems. The growth of the internet and social networks has introduced another valuable source of information for enhancing recommendation quality. Researchers have extensively investigated the information available on social networks, leading to numerous studies in this area [1].

### 2.2. POI data and its types

Adomavicius and Tujilin [8] have emphasized the fact that considering that the very broad scope of the POI concept, the focus of POI should be directly on areas related to RSs, including data mining, e-commerce personalization, databases, information retrieval, all-round POI systems, marketing and management systems. Various researchers have identified the need for defining and modeling of a POI in RSs through a consistent procedure. According to Verbert et al. [9], an accurate definition and modeling of POI makes it easy to identify what makes a POI and enables the reuse and exchange of POI data in application programs. Paper [58] show that trip recommendation for groups of tourists is a complex problem in tourism due to group dynamics and practical constraints. This paper proposes a novel approach to design personalized tours that satisfy all group members’ preferences while minimizing conflicts. Here, POIs in the tourism sector are also mentioned, which is very noteworthy.

- The different categories of POI and its types that can be used in RSs are described as follows:
- Physical: Physical POI shows the environmental positions of the user or the system in a specific location and includes features such as light, heat, and sound. According to Verbert et al. [9], physical POI has been widely investigated in home automation research.
- Computational: Computational POI has been extensively studied by Riki [10] and the Inclusive Learning Research Association [11]. Computational POI acquisition plays an important role in supporting intelligent agents that can select and recommend suitable resources for an RS.
- Location: Location POI has become very widespread in research [12,13]. Due to the increased use of mobile devices, location-based RSs have found a widely application [9].
- Time: Time POI consists of date and time information, which is usually based on minutes, hours, weeks, months, semesters, seasons, etc. Time is usually compound and is used in relation to other categories, e.g., location, in the form of time duration or time stamp. Time POI represents a sample or period during which POI information is needed or related to the user of the RS [9,14].
- User: A person who uses the recommender system or an individual for whom recommendation items are generated. For example, users in e-learning are e-learners who learn a learning model to produce accurate and valuable recommendations. Learning models have extensive access to user educational modeling and adaptive educational hypermedia [9]. User POI in the RS framework are likely to contain the user’s personal information, knowledge/performance, interests, and cognitive learning methods [15].
- Social relations: Social relations describe social circles, associations, communication, and dependence between two or more people. Social relations can be, for example, information about friends, people, neighbors, colleagues, enemies, and family. Some researchers have identified social relations as an important dimension of POI [9].

### 2.3. Fuzzy C-means clustering

Clustering is seen as an effective method for dealing with big data because it allows for entering the data space and identifying patterns in the data structure [16]. It is a type of unsupervised learning where samples are grouped together based on their similarities, forming clusters. A cluster consists of objects that are similar to each other but different from objects in other clusters [17]. Various criteria, such as distance, can be used to determine similarity, with objects that are closer to each other being grouped into clusters, known as distance-based clustering.

The available clustering methods can be classified into five categories: hierarchical, segmentation, density-based, network-based, and model-based methods [18]. Among the different methods for data clustering, the fuzzy K-Means (FKM) and C-Means (FCM) clustering methods have wide applications in different fields. K-Means algorithm is considered as one of the most famous methods for data clustering. In this algorithm, the data are classified into K different clusters after a number of iterations. One challenge discussed of the K-Means algorithm and most of the traditional clustering algorithms is that data belonging to each cluster is indicated by the number 0 or 1. In other words, in classical clustering, every input sample is assigned to a single cluster and cannot be classified into multiple clusters. In fact, the clusters do not overlap. The K-means algorithm, improved with genetic algorithms in [53], is a strong method for data classification. By combining the benefits of both approaches, it produces more accurate clusters and is less sensitive to the starting points.

[54] presents a new spiral optimization (SO) method for clustering. Unlike traditional SO, the proposed system divides the data into subgroups to enhance search diversity and improve clustering results. K-means is also used to enhance the algorithm. experiments show that this method also is good for clustering.

A fuzzy clustering algorithm was presented in [19] in order to address the challenges of the k-means clustering method. In Fuzzy clustering allows for a sample to be assigned to multiple clusters. In the fuzzy clustering algorithm, the belonging of each data to a specific cluster is determined by a real number inside [0,1]. The basic idea in fuzzy clustering is to assume each cluster as a set of elements. Then, by changing the definition of element membership in this set from a state where an element can only be a member of one cluster (partitioning state) to a state where each element can belong to different kinds of clusters with different degrees of membership, clusters that are more consistent with reality are presented [19]. In recent years, improved versions of this algorithm have been presented as well. To solve the problems caused by the initialization of clusters, Stetco et al. presented a new scheme in the initialization of clusters in the FCM clustering method [20]. The FCM method has wide applications in various fields, including remote sensing, time series clustering, color images segmentation, etc.

Another issue in clustering is finding the most appropriate number of clusters. In this study, the imperialist competitive optimization algorithm (which is explained in Section 2.4) is used to find a solution for this problem and make an initial guess to identify the number of clusters before running the algorithm.

### 2.4. Imperialist competitive optimization algorithm

The imperialist competitive optimization algorithm, which draws inspiration from imperialist competition, functions as an evolutionary algorithm designed to optimize a range of problems. Similar to other evolutionary algorithms, this algorithm begins by generating multiple random initial populations, referred to as countries. From these populations, the most exceptional elements are chosen as imperialists, while the remaining individuals are treated as the colony.

In order to presumed optimization issue, this algorithm evaluates N countries using a vector representation indicating their position in an n-dimensional space. The algorithm identifies the countries with the least cost according to the optimization function as imperialists, while the remaining countries are labeled as colonies [21]. To begin with, the algorithm calculates the normalized cost for each imperialist using the following method:


$$C_{n}=c_{n}-\text{max}\left\{c_{i}\right\}$$ (1)

In terms of relation (1), Cn represents the cost of the n-th imperialist after normalization. The highest cost among the imperialists is denoted as max{ci}, and cn is the cost of the n-th imperialist. Once the normalized cost is determined, the normalized relative power of each imperialist is computed, and this determines how the colonized countries are allocated among the imperialists.

Thus, the original count of colonies of a colonizer is equal to:


$$N\cdot C_{n}=round\left\{P_{n}\cdot \left(N_{col}\right)\right\}$$ (2)

where $N\cdot C_{n}$ denotes the original count of colonies of an empire and $N_{col}$ is the entirety of colonial countries in the population of the initial countries. The imperialist competitive algorithm commences by considering the initial state of all empires. the general policy in this algorithm is that, the imperialist countries pull the colonial countries to their side. As shown in relation (3), the total cost of each empire contains a part of the average cost of the colonies, in addition to the cost of the imperialist country.


$$C_{n}=cost\left(\text{imperialist}\right)\;+mean\left(cost\left(coloniesoimpire_{n}\right)\right)$$ (3)

The colony’s displacement is determined by the movement of the imperialist, resulting in a new position for the colony. The distance between the imperialist and the colony is denoted as “d,” and the colony’s movement is represented by the random variable “x,” which follows a uniform distribution.


$$x\sim U\left(\beta \times d\right)$$ (4)

In this equation β is a β>1 and close to 2. β =  2 can be a suitable choice. The existence of coefficient β>1 makes the colonial country approach it from different directions while moving towards the imperialist country. To increase the search area around the imperialist, an angular deviation equal to θ, with a uniform random distribution, is added to the main vector:


$$\theta \sim U\left(-\gamma .\gamma \right)$$ (5)

In equation (5), γ show a parameter that controls the range of angular deviation. In the next stage, there is a competition among imperialist powers. During this stage, the least strong colony of the weakest empire is given to a powerful empire, which may not necessarily be the strongest one. The selection of the empire is based on the likelihood of it being more powerful. Moreover, once an empire loses all its colonies, it is eliminated from the list of empires and becomes a colony for other empires in the imperialist competition. This process continues in a loop until a specified condition is met [21].

The suggested approach we employ is based on the imperialist competitive algorithm in order to derive the number of clusters and their centers. In fact, the values derived from this algorithm provide the different type of clusters and their centers to perform the fuzzy clustering.

## 3. An overview of previous studies

By analyzing user data and context, the RS can provide proactive, context-based suggestions, improving decision-making efficiency and reducing risks for businesses. Applications include hotels, hospitals, and banks. The RS considers factors like purchase history, item classification, and user location. Collaborative filtering algorithms are commonly used to analyze user data and provide recommendations [55].

[56] explores the use of digital nudges to promote sustainable tourism in personalized mobile guides. Given the growing emphasis on environmental sustainability, aligning user preferences with green travel behaviors is crucial. While changing travel habits can be challenging, digital nudges offer a promising approach. This study focuses on enhancing point-of-interest recommender systems to guide users towards sustainable itineraries. By combining personalized recommendations with information about the environmental impact of travel options, digital nudges can encourage users to choose more sustainable travel solutions.

In [57] authors introduce a novel decentralized collaborative learning framework for point-of-interest (POI) recommendation (DCLR). DCLR addresses privacy concerns by allowing users to train personalized models locally. To overcome data sparsity, the framework uses self-supervision signals and collaborative learning with attentive aggregation and mutual information maximization. DCLR demonstrates superior performance compared to existing on-device and centralized approaches, offering a more privacy-preserving and efficient solution for POI recommendation.

In [61] authors propose a novel POI recommendation method for LBSNs that effectively incorporates geographical influence. By using a spatial kernel weighting technique (CFSKW), the method addresses the limitations of existing approaches that often treat geography as a general probability distribution. Experimental results on two datasets demonstrate the superior performance of the proposed method compared to state-of-the-art techniques, especially in areas with uneven POI density.

In [62] Authors present an ontology-based spatial DSS for recommending entertainment and tourism centers in Arak, Iran. By leveraging domain-specific ontologies and real-time reasoning, the system provides personalized recommendations considering user preferences, location, time, and other factors. The proposed method outperforms traditional search engines and social media in finding relevant POIs, addressing the cold start problem by integrating with POI recommender systems. [63] proposes a novel travel location recommendation method that addresses the cold start problem. By combining convolutional neural networks and matrix factorization, the method can effectively recommend new travel locations based on their visual features. Experimental results on Flickr data demonstrate the superiority of the proposed method compared to existing approaches. [64] introduces Photo2Trip, a novel tour recommendation system that leverages visual features from geotagged photos. By integrating visual features into a probabilistic matrix factorization model, Photo2Trip effectively captures user interests and addresses data sparsity challenges. The system optimizes trip planning by considering user preferences, trip constraints, and visual features. Experimental results demonstrate the superior performance of Photo2Trip compared to existing methods, highlighting the effectiveness of visual features in improving personalized tour recommendations.

In the past, RSs used to produce recommendations only based on the user and item information, while the POI-based RSs use various POI information in addition to the user and item information to provide recommendations. Considering the method and time of using POI information in RSs, there are three different types of patterns [22]:

- POI pre-filtering: POI information is applied to the data as a filtering method before running the recommender process.
- POI post-filtering: POI information is ignored at the beginning of the process, and after the recommendations are produced based on the recommender model, the POI information is used to filter the results and provide recommendations based on the user’s interests.
- POI modeling: POI information is used directly in the recommender model.

There are numerous studies conducted on POI-based RSs and their applications in travel and tourism. In this section, some of these studies are presented based on the type of pattern, the type of POI information used, the applied POI, and the method used to generate recommendations. In [23], the authors proposed a smart tourism system based on ontology and POI, which provides tourists with appropriate services using the Internet of Things (IoT) technology and the semantic web services. The authors in [24] have reviewed the POI features of the user’s past travels based on each location. POI-based recommendations are inferred by finding users who are more similar to each other, a point is assigned to each location, and the recommendations whose locations do not meet the POI conditions are filtered and excluded. In [25], the researchers use the Latent Dirichlet Allocation method in order to extract the topics of tourists’ opinions, and consider the support vector machine (SVM) method to perform the sentiment analysis for them. Then, this data is processed along with the scores, place ratings, and historical information of users and places by the use of an artificial neural network (ANN), and the user’s favorite places that have been given less attention are recommended to the user. In [26], in this multi-level tourism RS, in addition to the user’s interests, his/her restrictions are considered as well. First, a number of cities are selected based on travel dates and tourists’ budgets. Then, a score calculated based on the users’ restrictions and interests is considered for each city, based on which suitable cities are recommended. The authors in [27] present a smart tourism system based on ontology and POI that offers tourists appropriate services using the IoT technology and semantic web services. In [28], the RS extracts the users’ profiles from TripAdvisor and processes the derived data set by the use of the SVM method. The aim of this system is to make the procedure easier and increase the precision of the recommendations provided to tourists based on open data. The authors in [29] propose a system consisting of a two-part network of traveler-hotel interactions using the neighbor-based link prediction method. They use this system in order to predict connections that do not exist on the network. In [30], the hybrid RS that is personalized based on social data, using the degree of similarity, trust and social relations among the tourists, recommends tourist attractions that are favorable to the tourists. In [31], hotel crowdsourced data, including official hotel information, multi-criteria ratings, and travelers’ textual comments, are extracted to create profiles for hotels and travelers using collaborative filtering. Then, based on the stochastic gradient descent (SGD) matrix factorization method, the initial recommendations are predicted, and after applying the POI information on them, the final recommendations are presented. In [32], the entropy-based mobility measure is used to classify photos that have a geographic tag, and gender is determined by face recognition in photos that are related to tourist tours. Then, a profile of users and tourist places based on gender is created, and tourist places are recommended by checking the users’ gender and their similarity. In [33], by taking advantage of the collaborative filtering method, tourists’ travel history and POI information, including weather conditions, time, and location, are used to recommend places in new cities that are similar to previous places of interest to tourists. In [34], tourist places are recommended based on a semantic approach using POI information. This method is based on the collaborative filtering algorithm and ranking of the POI information. In [59], Point-of-interest (POI) recommendation has become a significant research area in Location-Based Social Networks (LBSN). This paper proposes an LSTM-based method to address the data sparsity problem in POI recommendation. By using spatial binning to group nearby venues, our approach effectively leverages user preferences and historical data to suggest relevant POIs. Evaluations on real-world datasets demonstrate the superior performance of our method. Paper [60] introduces BERTSeqHybrid, a novel personalized sequential recommendation model for tourism route planning. BERTSeqHybrid overcomes the limitations of unidirectional models by utilizing bidirectional encoding and incorporating contextual data, asymmetric schemas, and topic modeling. To address the cold start problem, a novel user preference evaluation method based on explicit demographic data is proposed. Experimental results on Yelp and Flickr datasets demonstrate the superior performance of BERTSeqHybrid in terms of RMSE, F-Score, MAP. In [35], the dynamic topic modeling is used to derive the time-based distribution and implicit information of the user, and Additionally, High quantity of explicit data is extracted based on the entry and exit information, visual contents, and categories of tourists’ favorite places. be. Next, the collected data is utilized to identify similarities between users and locations by observing these similarities, matrix factorization is executed accordingly. Then, this information is used to detect user-user and location-location similarities, based on which the matrix factorization is performed. In [36], this location-based orientation-aware RS, which is designed in a hybrid way, uses the POI information of the users’ smart devices, in addition to their POI information, to recommend events using the IoT. It also uses information about virtual communities in social networks for when the user’s interests are not available based on the IoT data. In [37], the users’ emotional features in different places are determined, and the spatial and emotional POI information are combined to explore the user’s place of interest. Then, with the participation of factors of geographical distances and emotional similarity, the users’ favorite places are recommended. In [38], this model-based RS creates a profile for each place to recommend places to users based on them and the users’ interests. In this method, first, place tags extracted from the photo descriptions on Instagram are ranked using the TF-IDF, and then the photo description tags are classified using the Fast-Text method. The authors in [39] first present a data-driven method for exploring trips in location-based social networks, which examines how the users travel, and then it derives their travel patterns. It uses the extracted information for traveler clustering as well as distance clustering of travel destinations. In [40], using geotagged photos, the authors present a personalized travel RS based on the constrained matrix factorization method for multiple weighted information. Using the multiple information, a profile is first created for the users and travel locations, and for travel location recommendation, text, image, and distance data are assigned different weights based on their similarity. Then these weights are used to factorize the user-location matrix.

Compared to most of the methods presented in this field, the POI data of our proposed method, in addition to the location data that includes the geographic coordinates of the visited place, contains the temporal data, e.g., the time of day of the visit, the season of the visit, as well as the data related to the number of visits (popularity), weather conditions, user mood, traffic, and temperature, in order to increase the precision of recommendations.

The objective of the suggested approach is to create a model for the data utilizing the POI data. It involves integrating the imperialist competitive algorithm and the fuzzy C-Means method for clustering. The outcome of this process is then used to suggest appropriate visiting points to users. This methodology is centered around POI modeling.

## 4. Proposed method

**Fig 1:**
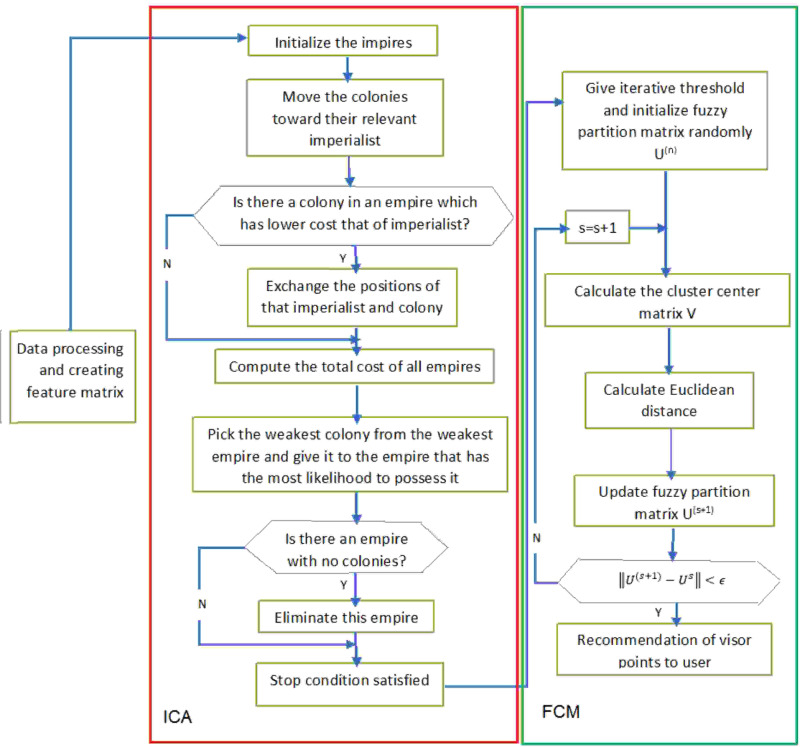
The proposed method.

The travel POI-based RS presented in this study is based on the fuzzy clustering algorithm and imperialist competitive optimization algorithm. Fig 1 illustrates the implementation process of the proposed method, which phase of which is explained separately in this section.

### 4.1. Extraction of POI information and creation feature matrix

The proposed method is evaluated based on the Flickr dataset, one of the largest online image and video sharing websites accessible to the public. The authors in [40] has emphasized the effect of geographic information and data on enhancement of the efficiency of RSs. It is because most of the tourist places are close to each other, e.g., 76% of the tourist places in Paris are less than 5 km away from each other. However, it should be noted that only geographical features and spatial distances are not important. For example, if a user who is in the city center and needs to rest is offered a nearby place based only on latitude and longitude, it may cause the user to be dissatisfied and not choose the recommended place. Thus, in order to provide more accurate recommendations, other features should be considered as supplementing information, in addition to geographic tags [37].

To extract the POI information required for the proposed method, the tags shown in Table 1 have been extracted from the site for each visited location. To extract the features, we used ResNet50, which has the advantage of improving the problem of overfitting. Therefore, we used ResNet50 as the foundation model, which was trained on the dataset. In our TrFEMNet architecture, we established the General Feature Extraction Module (GFEM) by keeping the weights of the lower layers of ResNet50 fixed. The upper layers were then trained again using the target data samples to enable the Specific Feature Extraction Module (SFEM). The features from both the GFEM and SFEM were combined and inputted into the Projection Head (PH) module. The output from the PH module was then processed by the Softmax classifier to produce the final output.

**Table 1:** Features of the visit point.

Feature	Description
X (Lat)	X geographic coordinates of the visit point
Y(Lon)	Y geographic coordinates of the visit point
Views:	The number of visits to the location of interest (popularity of the location)
Current Weather	The weather condition at the time of taking the photo
Time Of The Day	Visiting time during the day
User Mode	The user mood during the visit
Season	The season of visit
Traffic	The traffic volume during traffic time

The PH module, which includes a two-layer MLP with ReLu nonlinearity, is used to extract features at various levels in the hierarchy. This helps to improve the performance of the classifier by providing a more effective representation. Additionally, dense layers were added on top of the convolution layers. To compare with TrFEMNet, three other models were constructed without the use of the PH module. The number of layers in these models varied, with some having fixed weights and others having trainable weights.

**Fig 2:**
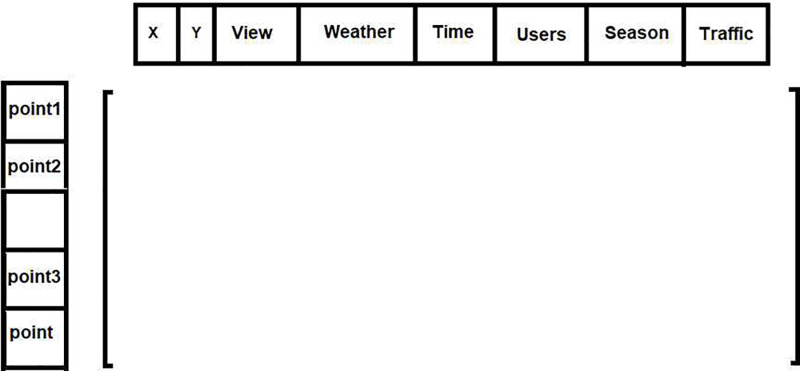
Structure of feature matrix based on POI information.

In this research, ResNet50 is used as the basis for developing models, with Softmax employed as the activation function in the output layer. The models can be characterized as described below. Model 1 includes a dense layer with 1024 trainable neurons, not counting the final dense layer for classification. In this model, all the convolutional layers of ResNet50 are kept fixed, and only one dense layer is allowed to be trained. Model 2 bears resemblance to Model 1, with the sole difference being its composition of two trainable dense layers, sized 1024 and 512. In model 3, only the final convolution layer is not fixed while the rest remain unchanged. Since ResNet50 has a total of 48 convolutional layers, 47 of them are kept fixed and one convolutional layer along with a dense layer consisting of 1024 neurons are utilized for training. In regards to the TrFEMNet model, we maintain two convolution layers and two dense layers with 1024 and 512 neurons for SFEM and utilize them as the fixed layers for GFEM. The results from GFEM and SFEM are then directed to PH and subsequently to the classifier. The features extracted for each POI from the visit include: location longitude and latitude, number of visits (popularity rate), weather condition, time of day, user mood, traffic volume, season, and temperature. Thus, the spatial POI data includes geographic longitude and latitude, and the temporal POI data includes season and time of visit. The features of the number of visits to location of interest, weather condition, traffic volume, and user mood are used to enhance the precision of the suggested approach and provide more accurate recommendations. The structure (rows and columns) of the matrix related to POI data is shown in Fig 2.

### 4.2. Determination of number of clusters using ICA

To cluster different places, similar places are first placed in a cluster using the ICA and based on the different POI features of the places. Therefore, without the It is necessary to determine the exact number of clusters beforehand, the visited places are automatically clustered. As described in Section 2.3, countries are first initialized and their imperialists and colonies are then determined. Then the colonial countries are randomly moved towards their imperialists, and if the power of the colonies is greater than the corresponding imperialists, the colony with more power is known as the new imperialist, and the old imperialist is considered as its colony as well. Next, the imperial power with the least sturdy colony is paired with another imperial power in a random manner. However, in cases where there is an imperial power lacking a colony, it is left out from the process. The mentioned procedure is carried out until the specified requirement is met. Algorithm 1 displays the pseudocode for the ICA.

**Algorithm 1:** The pseudo-code of ICA.

The pseudo-code of the Imperialist Competitive Algorithm Input: Numpopulation, NumInitialImperialists Output: Best Imperialists initialize population randomly For i = 1 to Numpopulation Compute the evaluation cost ci Arrange the calculated cost ci in a descending order. Choose a group of NumInitialImperialists individuals from the total Numpopulation Evaluated and standardized the expense of each Imperialist country cn Calculate the normalized power for each Imperialist pn. Allocate the remaining nations to the Imperialists and designate them as Numcolonies. EndFor For j = 1 to NumInitialImperialists Shift the settlement in the direction of the applicable Imperialist agenda of assimilation. Calculate the expenses of countries that have been integrated Carry out a revolution in the recently established colony. If the expense of establishing a new colony is lower than the expense of imperialis Next, switch the positions of colony and Imperialist. Choose the most fragile colony from the least powerful empire and transfer its ownership to the empire with the highest probability of acquiring it. EndFor Imperialist with no coloniesIf there is remive the ImperialistsThen Until stopping condition is reached

Our proposed method used ICA in order to derive the number of clusters and their centers and cluster the places based on their POI features. The authors in [41] presented an improved ICA, which has a better performance than the classical ICA in terms of clustering. Their ICA is used in our proposed method. The goodness of a clustering result can be established based on clustering evaluation criteria corresponding to mathematical-statistical functions.

A clustering evaluation criterion follows two goals: 1) Determining the number of clusters 2. Identifying the best clustering mode based on the number of clusters. Each clustering evaluation criterion should consider the following two aspects of clustering: 1) Continuity or compactness: The goal is to make patterns within a cluster as similar as possible. The dispersion or variance of patterns in a cluster indicates how compact and continuous the patterns are within the cluster. 2. Separation: Clusters should be as far apart as possible. The distance between the centers of the clusters (e.g., the Euclidean distance) may represent the separation of the clusters [42].

There are various evaluation criteria for evaluation of non-fuzzy clustering, including PBM(Pakhira,Bandyopadhyay,Maulik), DI (Dunn Index), DB(Davies & Bouldin) [43], CS(Chou, Su, Lai) [44] criteria can be mentioned. For all these criteria, their maximum or minimum values indicates the optimal clustering of the set of patterns/data. Therefore, they can be used in meta-heuristic optimization algorithms. The computational complexity of the DI criterion increases greatly as the number of clusters and the data size increase. The PBM criterion is used more in cases where the number of clusters is small and the data has low dispersion and high density. In addition to having an acceptable computational complexity in large data sets and hierarchical data sets, the DB criterion can also provide good results for data with different dimensions. The CS criterion performs effectively for clusters with different densities. Thus, this study uses the DB and CS criteria in order to evaluate clustering. The evaluation results of these criteria are given in Section 5.5.

This evaluation criterion for databases is determined by comparing the dispersion within a cluster to the dispersion between clusters. The dispersion within a cluster is calculated for cluster i, and then the distance between cluster i and cluster j is also calculated using equations (6) and (8) [43].


$$S_{i.q}={\left[\frac{1}{x}\sum \vec{x}-{\vec{m}}_{2}^{q}\right]}^{\frac{1}{q}}$$ (6)

$$D_{ij.q}={\left\{\overset{d}{\underset{p=1}{\sum }}{\left|m_{i.p}-m_{j.p}\right|}^{t}\right\}}^{\frac{1}{t}}{\vec{m}}_{i}-{\vec{m}}_{j}$$ (7)

where mi denotes the center of the i-th cluster, t ≥  1, and q and t can be set independently. Ni denotes the number of patterns belonging to cluster Ci. Dij,t calculates the t-th norm for the centers of cluster Ci (mi) and cluster Cj (mj). The value of the DB evaluation criteria is calculated using relations (8) and (9).


$$R_{i.qt}=\underset{j\in K.j\neq i}{\max}\left\{\frac{S_{i.q}+S_{j.q}}{D_{ij.t}}\right\}$$ (8)

$$DB\left(K\right)=\frac{1}{K}\overset{k}{\underset{i=1}{\sum }}R_{i.qt}$$ (9)

In relation (10), K represents the K-th cluster. The lowest value derived for the DB criterion indicates the optimal clustering.

CS evaluation criterion: Before calculating the CS evaluation criterion, the determination of the center of every cluster using the average patterns of that cluster based on relation (10) [44].


$${\vec{m}}_{i}=\frac{1}{N_{i}}\underset{x_{j\in C_{i}}}{\sum }{\vec{x}}_{j}$$ (10)

where Ni denotes the number of patterns belonging to cluster Ci. The distance measure between the two samples $\vec{X_{i}}$ and $\vec{X_{j}}$ is represented by $d\left(\vec{X_{i}}.\vec{X_{j}}\right)$. The CS criterion is defined as relation (11).


$$CS\left(K\right)=\frac{\sum_{i=1}^{K}\left[\frac{1}{N_{i}}\sum_{{\vec{X}}_{i}\in C_{i}}\underset{{\vec{X}}_{q}\in C_{i}}{\max}\left\{d\left(\vec{X_{i}}.\vec{X_{j}}\right)\right\}\right]}{\sum_{i=1}^{K}\left[\underset{j\in K.j\neq i}{\min}\{d\left(\vec{m_{i}}.\vec{m_{j}}\right)\right]}$$ (11)

Similar to the DB evaluation criterion, the CS criterion is equal referring to the proportion of the distance between clusters intra-cluster distance to inter-cluster distance; hence, it should be expressed as the minimum denominator and the maximum denominator, and the total value of CS should be minimized.

Basically, the passage is saying that in each step of the algorithm, the distance between each sample and the center of the cluster is measured. The sample is then assigned to the cluster with the shortest distance. The improved ICA is then used on the cluster centers and this process is repeated until the best number and centers of the clusters are found.

Considering that the number of clusters is not specified by the user at the beginning, the answers should be expressed in such a way that the number of clusters can be derived at the same time as the data is clustered at runtime. Therefore, assuming that the maximum number of clusters is equal to K and the number of dimensions or features of the data set is equal to d, then each of the possible solutions would be a matrix with dimensions k × (d + 1); and to get the optimal number of clusters, a decision variable is added to the existing decision variables to solve the clustering problem. In other words, each cluster center has a decision variable called the activation decision variable, which determines whether the corresponding cluster is active or inactive. The allowed values for the activation decision variable are real numbers inside the interval (0,1). A cluster center is activated if the value of the corresponding decision variable is greater than 5.0; otherwise, the cluster center is deactivated. To evaluate the answers in the ICA, any of the clustering evaluation criteria can be used. In this study, two clustering evaluation criteria, i.e., DB and CS, were considered as the objective function according to relation (12) in order to minimize the value of the defined CS and DB criteria.


$$f_{1}=CS(K)f_{2}=DB(K)$$ (12)

###  4.3. Fuzzy clustering of data

In C-Means clustering phase, let the set of visiting locations is denoted by *L= [l*_*1*_*.l*_*2*_ *....l*_*n*_*]* and the set of clusters is represented by $C=\left[c_{1}.c_{2}\ldots .c_{c}\right]$. Each element of the set L can be a value between 0 and 1 as the degree of membership in more than one cluster. Thus, considering the fact that each visit location is placed in more than one cluster, one of the challenges of RSs, i.e., data privacy, is solved to a large extent, and there would be more neighbors for the visit locations of each cluster. The pseudocode of the fuzzy clustering algorithm is presented in Algorithm 2.

**Algorithm 2:** Pseudocode of fuzzy clustering algorithm.

The pseudo-code of the Fuzzy C-Means (FCM) Input: L and C. Output: final Fuzzy C-Means clusters. 1: Select an initial fuzzy pseudo-partition, i.e., assign values to all ui,j 2: Repeat 3: Find the average position of the data points within each cluster by using the fuzzy partitioning method. 4: Update the fuzzy partition, i.e, the ui,j. 5: Until the centroids remain the same

As can be seen in the pseudo-code of Algorithm 2, if L is the set of visited locations, C is the set of c clusters, and i and j are considered the indices of each of the clusters and locations respectively, first the degree of belonging of each location to the clusters based on ui,j is derived and calculated and then the center of each cluster is determined. The locations are re-clustered Based on the new centers. This is repeated until the changes of the cluster centers are smaller than a certain threshold, and finally the final clusters are derived.

The fuzzy clustering algorithm requires a predetermined number of clusters (c) to be specified beforehand. The algorithm defines an objective function as stated in reference [19].


$$J=\overset{c}{\underset{i=1}{\sum }}\overset{n}{\underset{k=1}{\sum }}u_{ik}^{m}x_{k}-v_{i}^{2}$$ (13)

represents the k-th sample, Vi represents the center of the i-th cluster, and n represents the count of visited locations. uik represents the degree of belonging of the i-th sample to the k-th cluster. The symbol ||*|| x denotes the degree of similarity (distance) of the sample with (from) the center of the cluster, for which any function that expresses the similarity of the sample to the center of the cluster can be used. From uik, a matrix U with c rows and n columns can be defined, with its components being any value between 0 and 1. If all the components of the matrix U belong to {0,1}, the algorithm would resemble the conventional C-Means algorithm. While the elements in the matrix U can have values ranging between 0 and 1, the total of all elements in each column must add up to 1, thus:


$$\overset{c}{\underset{i=1}{\sum }}u_{ik}=1.\forall k=1.\cdots .n$$ (14)

This requirement implies that the total membership score of every sample in cluster c should add up to 1. By adhering to this condition and minimizing the objective function, we can achieve this:


$$v_{i}=\frac{\sum_{k=1}^{n}u_{ik}^{m}x_{k}}{\sum_{k=1}^{n}u_{ik}^{m}}$$ (15)

$$u_{ik}=\frac{1}{\sum_{j=1}^{c}{\left(\frac{d_{ik}}{d_{jk}}\right)}^{2/\left(m-1\right)}}$$ (16)

### 4.4. Recommending visiting points to user

After the centers and number of clusters are determined using the ICA, the centers are considered as the initial centers for the FCM algorithm. Then the FCM algorithm is run, and finally the clustered locations are derived. Then, the clusters that belonging to user locations are determined for each user. For example, the locations visited by a user may belong to clusters 1, 2, and 4. The cluster with the most locations visited by the user is considered as the user’s favorite cluster. Then, the locations belonging to that cluster are used to predict the next locations for the intended user. To this end, the locations belonging to the cluster are sorted based on the closest location to the center of the cluster. Then, the locations that have been visited by the user and belong to that cluster are excluded. And finally, from the rest of the locations, some are recommended to the user based on the number of required places.

The choice of the ICA algorithm is beneficial because it can accurately determine the exact number of clusters and their centers. Finding the optimal number of clusters has always been a challenge in clustering algorithms, where elements within a cluster should have the highest similarity while having the maximum difference from other clusters. Some methods randomly determine the number of clusters or perform sensitivity analysis. However, accurately determining this number is crucial, which ICA can accomplish. Determining cluster centers is also very critical. Some algorithms select them randomly, while others try to make a more accurate initial selection. We perform this task precisely at the beginning using the ICA algorithm. After this stage, the results of this stage, namely the number of clusters and their centers, are sent to the FCM algorithm. Due to its fuzzy nature, this algorithm is highly compatible with the nature of tourist site selection. A tourist’s choice of a place to visit is not absolute; for example, choosing to visit historical sites does not mean a hatred of swimming and using the beach. Therefore, tourists have a percentage-based tendency to be present in tourist locations, which is highly compatible with the fuzzy nature of clustering tools.

## 5. Methods

In this section, we introduce data set used to evaluate the proposed method, present the data pre-processing and the parameter setting approaches, the evaluation results, and finally compare the results with those of other similar methods.

### 5.1. Dataset

To ensure the ethical and legal use of the data, we confirm that all data collection and processing procedures adhered to the terms of service and privacy policies of Flickr. No personal information was used in this study.

Flickr is a widely used platform for sharing images and videos on the internet and offers various online services and online communities. It was founded by Ludicorp in 2004 and acquired by Yahoo in 2005. This site has gradually turned into an image-based social network [45]. To evaluate the proposed method, this study uses a dataset derived as follows: a crawler retrieves a set of visited locations in America and mainly in Europe in the period from 2015 to 2019, and collects the tags of the geographical coordinates of the locations, number of visits (popularity rate), weather condition, time of day, user mood, traffic volume, season, and temperature. Table 2 presents the extracted tags for each visited location.

**Table 2:** Values of ICA parameters.

Parameter	Value
Number of countries	100
Number of initial imperialists	8
B	2
γ	7.0
ε	05.0
Number of iterations	200

### 5.2. Data pre-processing and parameter setting

In order to unify the features and data cleaning operations, out of the 10,000 records, 2,500 records that included all the features have been used for simulation. For each record, 20 visited locations have been specified. Out of these 20 locations, 15 are considered for training and 5 for testing the proposed model. In other words, using 15 visited locations, 5 locations are predicted for each user. The features of the used dataset are presented based on numerical and non-numerical values. In order to use the non-numerical values of some features, mapping is used to quantify the non-numerical features with numerical values, as specified in relation (17):


$$NormalizedValue=\frac{\max\left(field\right)-\text{min}\left(field\right)}{number}$$ (17)

where field denotes the value of the desired feature, number denotes the number of ranges considered for mapping non-numerical values, and Normalized Value represents the correct and normalized value of the desired feature. In fact, the values of all the features have been converted to the integer values for the purpose of normalization. For example, the values of the weather condition (sunny, cloudy and rainy, snowy and cold, and humid and moderate) are mapped to values from 1 to 4, the time of visit during the day (morning, noon, evening, and night) to values from1 to 4, the user mood (normal and natural, happy, nervous, upset, excited, tired and worried) to values from 1 to 6, and the season and the traffic volume to the integer’s numbers in the same way. After performing pre-processing on the initial dataset, a new data set is derived.

As mentioned in section 4.2, the implementation of the ICA requires determination of a series of initial parameters, e.g., β, *γ*, *ε* that must be specified, population size (number of countries), number of initial imperialists, the number of iterations. These values are presented in Table 2.

## 6. Evaluation

### 6.1. Evaluation criteria

In order to assess the suggested approach, the precision, mean absolute precision, and mean absolute error (MAE) are utilized as evaluation metrics. The precision criterion involves calculating the ratio of accurate predictions to the overall number of predictions, as determined by the formula (18) [1].


$$precision=\frac{\text{number of items that are relevent}}{numberofallrecommendeditems}$$ (18)

The MAP criterions is also calculated via relation (19):


$$MAP=\frac{1}{N_{q}}\overset{N_{q}}{\underset{i=1}{\sum }}AP_{i}$$ (19)

In this equation Nq denotes the number of tested queries and APi is the mean precision for the i-th query. APi is calculated based on the relationship $\frac{1}{r}$, where r denotes the rank of the desired item in the sorted list of items. In fact, the criteria of precision and MAP represent the quality of recommendations provided, and the higher their value, the more proficient the recommender system becomes in offering accurate recommendations.

Another approach to evaluate the results of RSs is to check the error rate of recommendations provided to users. Different studies have provided different relations for calculation of the error value. One of the simplest and most basic relations has been proposed in [46], which, instead of considering the numerical values of the predicted ranks and the actual ranks, considers their values in binary form and evaluates each recommendation in two useful and non-useful modes. In this study, two modes of visited or non-visited are considered for the recommended locations. The MAE criterion is calculated based according to relation (21), where the amount of Accuracy is according to the number of the user’s recommendations on interest compared to the total recommendations [46]:


MAE=Accuracy (20)

### 6.2. Evaluation results of the proposed method

**Fig 3:**
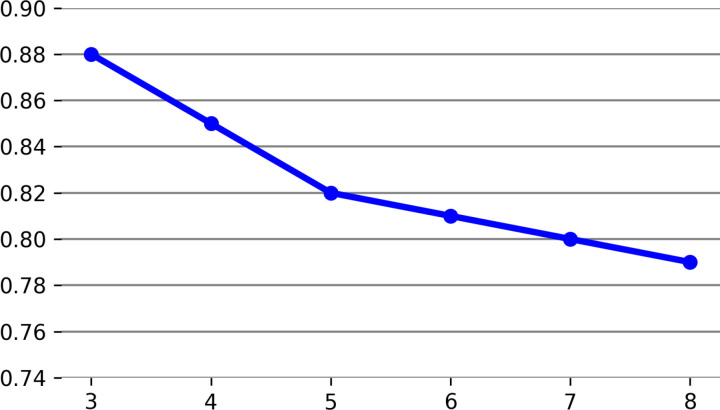
Values of precision criterion for the FCM clustering algorithm based on the number of different clusters.

The first step in assessing the proposed method involves examining the accuracy of the clustering process for the FCM method using varying numbers of clusters. Then, the criteria of the ICA are evaluated based on the DB and CS factors. The values of precision criterion are listed in Table 3 based on the count of different clusters for the FCM clustering algorithm, and the value chart is shown in Fig 3.

**Table 3:** Precision values based on the number of different clusters for the FCM algorithm.

Number of clusters	precision
3	88.0
4	85.0
5	82.0
6	81.0
7	7.0
8	79.0

**Fig 4:**
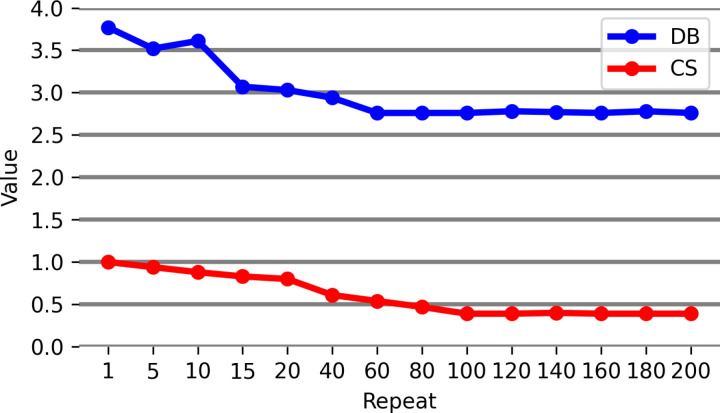
Comparison of DB and CS clustering evaluation indices.

For the FCM algorithm, the highest precision is derived with the number of 3 clusters. Regarding the effective criteria in the ICA, the evaluation results indicate that the DB criterion is more suitable than the CS criterion and has a better function in determination of the number and centers of clusters. The CS criterion has a relatively higher computational complexity compared to the DB, and the evaluation results in [47] also indicate that the DB criterion outperforms the other clustering evaluation criteria. Fig 4 shows the changes of these two criteria in different iterations.

### 6.3. Comparison of the proposed method with existing methods

This section evaluates the proposed method in comparison to other existing methods used in travel point of interest POI-based RSs. The goal is to highlight the advantages of the proposed method compared to previous studies in this domain.

Initially, the proposed technique is evaluated alongside several commonly employed basic approaches, such as Popularity Rank (PR) [48], Classic Rank (CLR) [49], and Frequent Rank (FR) [50], which are frequently utilized in various research studies to assess precision. It should be mentioned that all these basic methods have been implemented and evaluated with the dataset presented in this study. Then, a number of previous studies conducted on travel RSs based on geo-tagged photos extracted from Flickr [24,32–34] are evaluated in order to compare them with the proposed method. The evaluation criteria in all of the studies include the parameters related to the recommendation quality, e.g., precision, MAP, and MAE.

Table 4 presents the precision, MAP, and MAE the amount of the proposed method in comparison with those of the other basic methods. To evaluate all the methods, 2500 records of the Flickr dataset for 100 users were extracted from the dataset used in this study. To assess the proposed method’s efficiency, its outcomes were compared against the four fundamental methods detailed in this section.

**Table 4:** Precision, MAP, and MAE values of the proposed method in comparison with other basic methods.

Method	precision	MAP	MPA
Proposed	0.89	0.73	0.11
PR	0.72	0.59	0.28
CLR	0.69	0.61	0.31
FR	0.75	0.64	025

Popularity Rank (PR): In this method, locations are ranked based on the public popularity level. The popularity of each place is determined by counting the number of unique visits to the preferred locations [48].

Classic Rank (CLR): This method does not consider all users the same, and considering the HITS-based inference, it calculates the tourist attractions of the locations and users’ travel experiences as a credit score for them. It also extracts the central score from the data related to the interactions between users and locations [49].

Frequent Rank (FR): This approach involves utilizing the PrefixSpan algorithm to identify frequent locations and sequential patterns with frequencies equal to or greater than the minimum support threshold value [50].

Based on the findings, the proposed method outperforms the other basic methods due to the use of POI data based on time, location, weather conditions, temperature, user mood, traffic volume, as well as the detailed analysis and data clustering. In fact, the proposed method provides users with detailed recommendations by examining the history of users’ past visits.

A comparison of the proposed method with similar studies based on the parameters of precision, MAP, and MAE is presented in Table 5. As mentioned earlier, all the methods have used Flickr datasets collected in different locations.

**Table 5:** Precision, MAP, and MAE values of the proposed method compared to other studies.

Title of study	precision	MAP	MAE
Suggesting travel destinations based on location-tagged pictures shared on social media platforms for tourists.	83.0	71.0	17.0
Extracting information from location-based social networks to categorize travelers and destinations through mining expeditions.	86.0	62.0	14.0
Personalized travel destination recommendations that are tailored to individual preferences based on Weighted Multi-Information Constrained Matrix Factorization	81.0	52.0	19.0
The technique of recommending travel locations based on gender-aware and similarities, by analyzing geotagged photos	80.0	69.0	20.0
Proposed method	89.0	73.0	11.0

In order to assess the efficiency of the suggested approach, its outcomes were contrasted with the four fundamental methods delineated in this section.

As can be seen in Table 5, the precision and MAP values of the proposed method are higher than those of the other methods and the MAE values are lower compared to the other methods. Using several different POIs and combining the two fuzzy clustering and imperialist competitive optimization algorithms before giving the final recommendation in the proposed method increases the precision and decreases the error, compared to the values obtained in [24], which uses a smaller number of POIs and is only based on the K-Means clustering algorithm. In addition, compared to the methods used in [39] and [33], which recommend trips based on the users’ travel preferences inferred from their previous trips as well as using the collaborative filtering approach, the proposed method is more accurate, because the proposed method has processed more complete and comprehensive information of a visit, based on which it performs clustering and provides more accurate recommendations. In the method used in [32], locations are classified based on tourists’ gender, and less POI information is used compared to the proposed method. The main difference between our proposed method and the methods presented in other studies is the addition of more POIs (weather condition, user mood, traffic volume, etc.). While only time and location POIs have been used in many similar studies.

## 7. Conclusion

This study recommends new places for the user to travel, considering the POI data and based on users’ history of trips and visits to different locations. The proposed method uses geo-tagged photos that are available in social media in order to generate location recommendations according to users’ interests and visiting positions. In this way, it is able to understand location, time, date, weather, temperature, and user mood. This method is based on the combination of the FCM clustering and ICA, which are used to identify clusters of tourist locations. Travel histories are extracted from geotagged photos. The proposed method was evaluated and compared with some travel destination recommendation methods using the photo collections that are publicly available on the Flickr. According to the evaluation results, this method is able to provide location recommendations based on users’ interests and current location of their visits. The proposed method is suitable for travel recommendations due to considering the similarities between the users (user-user similarity model) based on their trip. Based on the finding in this research about the proposed method, using the power of social influence and POI data for location-sensitive items is an effective approach for travel recommendation. In fact, using the combining the FCM clustering algorithm and ICA, the proposed method increases the precision of recommendations. Also, by the use of personalized services with more POI data (location, number of visits, weather condition, time of the day, user mood, traffic volume, season, and temperature), the RS error decreases and more accurate recommendations are provided.

Suggestions for future research focus on expanding the understanding of influencing factors. Exploring the impact of social factors, such as user social relationships, opinions of friends, and influencers on recommendations, as well as considering psychological factors like user interests, lifestyle, and personal preferences can be impactful. Additionally, investigating the effects of environmental factors such as air pollution, area safety, and accessibility to public facilities on location selection as environmental factors can be influential. Furthermore, developing deep learning models, utilizing recurrent neural networks (RNNs) to model time series and better understand user preferences based on their interaction history, can yield more accurate results. Attention models can also be employed to focus on important data features and improve model accuracy. Developing hybrid models, combining deep learning models with traditional methods to leverage the advantages of both approaches, is also noteworthy. Evaluating the model on larger and more diverse datasets to assess its generalizability, evaluating in various scenarios such as group trips, solo trips, and budget-constrained trips, and conducting qualitative studies to assess user satisfaction with system recommendations can also be part of future work. Enabling users to provide feedback on recommendations and continuously updating the model, as well as combining content-based, collaborative, and knowledge-based recommendations to provide more diverse and accurate recommendations, are also crucial.

This research represents a significant step towards developing travel recommendation systems. However, there are still ample opportunities for further research and system improvement. Given the growing importance of tourism and the role of technology in enhancing travel experiences, investing in this area can lead to new opportunities and improve people’s quality of life.

## Supporting information

S1 Data DataSet. (TXT)
